# Long‐term exposure to high altitude attenuates verbal and spatial working memory: Evidence from an event‐related potential study

**DOI:** 10.1002/brb3.1256

**Published:** 2019-03-20

**Authors:** Hailin Ma, Delong Zhang, Xuebing Li, Huifang Ma, Niannian Wang, Yan Wang

**Affiliations:** ^1^ Center on Aging Psychology CAS Key Laboratory of Mental Health Institute of Psychology Beijing China; ^2^ Plateau Brain Science Research Center Tibet University/South China Normal University Guangzhou/Tibet China; ^3^ Center for the Study of Applied Psychology, Key Laboratory of Mental Health and Cognitive Science of Guangdong Province, School of Psychology South China Normal University Guangzhou China; ^4^ College of Management Tianjin University Tianjin China

**Keywords:** event‐related potential, high altitude, *n*‐back, working memory

## Abstract

**Introduction:**

This study aimed to determine the neurocognitive basis underlying the effects of long‐term high‐altitude (HA) exposure on working memory (WM).

**Methods:**

Using event‐related potentials (ERPs), we compared the performance of an HA group (individuals who had lived at HA for 3 years but were born and raised at low altitude [LA]) to that of an LA group (individuals who had only lived at LA) on verbal and spatial *n*‐back tasks (i.e., 1‐ and 2‐back memory load).

**Results:**

Response accuracy of the HA group was significantly decreased in comparison to the LA group in both the verbal and spatial 2‐back tasks. The P2 amplitude was larger in the HA than in the LA group in the spatial, but not the verbal 2‐back task. A smaller late‐positive potential (LPP) amplitude was found in the HA group in both the verbal and spatial 2‐back tasks.

**Conclusions:**

These results suggest that HA impairs the matching (P2) process in spatial WM tasks and the maintenance (LPP) process in both verbal and spatial WM tasks, indicating that HA had a different effect on verbal and spatial 2‐back task performance.

## 1 INTRODUCTION

Oxygen is essential for maintaining normal human brain function; therefore, the most important and influential aspect of living in high‐altitude (HA) areas is hypoxia. Chronic exposure to HA leads to deficits in cognition such as in attention, memory, and executive function (Virués‐Ortega, Garrido, Javierre, & Kloezeman, 2010; Yan, 2014).

As a core cognitive capability, working memory (WM) may be influenced by HA conditions. The prefrontal cortex, one of the critical brain regions involved in WM, shows decreased gray matter volume and regional cerebral glucose metabolism in HA residents (Zhang, Yan, Gong, & Weng, 2011). In addition to verbal and spatial WM being impaired in people born and raised at HA, previous behavioral studies (Yan, Zhang, Gong, & Weng 2011a, 2011b) suggest that short‐term exposure to HA leads to decreased performance in several WM‐related cognitive tasks. This was also seen in a psychological assessment of college students who originally resided at near sea level (Yan, 2014). The neurocognitive basis underlying WM deficits due to chronic hypoxic stress remains unclear, and its study may offer a unique perspective into the functional/adaptational changes that occur in the brain at HA.

WM entails multiple mental components, including the form‐specific information storage systems composed of a phonological loop that processes verbal information, a visuo‐spatial sketchpad that processes spatial information, and a central executive system comprising three cognitive processes (inhibition, shifting, and updating) (Baddeley, 2003). Verbal and spatial WM are both reportedly impaired in HA residents (Yan et al., 2011a, 2011b); however, the impact of HA on the executive system has not been elucidated. Previous study using the Go/NoGo and the Trail Making Test (part B) tasks showed that inhibition and shifting processes were affected in HA residents (Ma, Wang, Wu, Luo, & Han, 2015; Rimoldi et al., 2016). It remains unclear whether the updating process is involved in WM impairment. Therefore, we decided to use the *n*‐back task to further identify and locate the specific neurocognitive components of the updating process in WM that are affected in populations under chronic hypoxic stress.

Event‐related potentials (ERPs) have high temporal resolution and can provide insight into the nature and precise temporal sequence of brain processes. The association between ERP components and related mental activities is explicit, rendering these components appropriate tools to determine which WM elements are affected by HA exposure. According to previous studies, specific ERP components are related to each of the three cognitive processes involved in the *n*‐back task. The occipital N1 components are related to attention toward and the encoding of stimuli (Luck & Kappenman, 2011). The P2 component, located in the frontal area, is related to top‐down processing, matching sensory input information with memory representations (Evans & Federmeier, 2007; Federmeier & Benjamin, 2005; Luck & Hillyard, 1994). The parietal late positive potential (LPP) is related to the attentional demands of WM tasks (Corbetta, Miezin, Shulman, & Petersen, 1993; Owen, McMillan, Laird, & Bullmore, 2010; Yantis et al., 2002) and within the memory system has been shown to reflect sustained processing and maintenance of information (Ruchkin, Johnson, Canoune, & Ritter, 1990; Weinberg & Hajcak, 2011).

To determine the modulation effect of HA on cognitive function, numerous studies have focused on native residents (e.g., indigenous Tibetans) (Richardson et al., 2011; Wu & Kayser, 2006) or individuals with acute exposure to HA (Hayashi, Matsuzawa, Kubo, & Kobayashi, 2005; Thakur, Ray, Anand, & Panjwani, 2011). However, few studies have directly examined individuals who were born and raised at low altitudes (LA), but relocated to an HA environment for an extended period. Notably, investigation of the effect of HA on the cognitive function of long‐term immigrants will help evaluate impairment due to long‐term HA exposure and elucidate the dynamic adaptation to HA, with considerable social and economic value. In this study, we aimed to use ERPs to explore the cognitive aspects of WM of healthy young people who were born and raised at LA but experienced hypoxia exposure for 3 years due to HA. We applied spatial and verbal *n*‐back paradigms to measure WM performance of such individuals compared to individuals who were born and lived in LA areas. We used response time (RT) to reflect processing efficiency and accuracy rate to measure performance effectiveness. The relevant ERP components were examined to explore any underlying changes in mental processes.

## 2 METHODS AND MATERIALS

### 2.1 Participants

Forty healthy, right‐handed college students (19 males; aged 20–24 years) were recruited. All participants were of Han ethnicity, with normal or corrected‐to‐normal vision. The HA group included 19 individuals (10 males; aged 22.26 ± 1.24 years) who were born and raised (i.e., at least 18 years) in an LA location (<1,000 m) and had lived at HA (Lhasa, 3,650 m) for 3 years. The LA group included 21 participants (nine males; aged 22.05 ± 1.60 years) who had never lived in an HA area. Notably, SpO_2_ was measured using a pulse oximeter (CMS50D; CONTEC, Qinhuangdao, China) at resting state with a warmed hand and differed between the HA and LA groups [*t* (1, 40) = −12.47, *p* < .001], being 90.71 ± 2.31% and 97.48 ± 0.93% in the HA and LA groups, respectively. The data of the HA group were collected at a laboratory at Lhasa University (3,650 m) and the data of the LA group were collected at the Institute of Psychology at the Chinese Academy of Sciences in Beijing (43.5 m).

Notably, college entrance scores were matched between the two groups (*p* > 0.05). The experiment was conducted in accordance with the Declaration of Helsinki and was approved by the Ethics Committee of the Institute of Psychology, Chinese Academy of Sciences. All participants signed an informed consent form before the experiment.

## 3 STIMULI AND PROCEDURE

**Figure 1:**
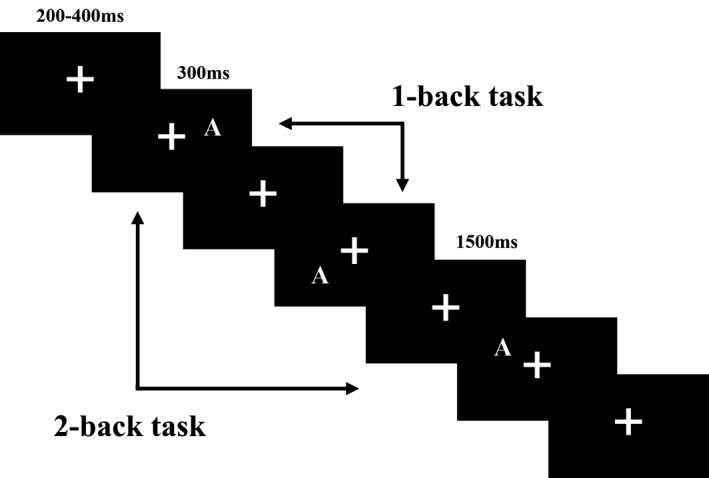
Stimuli and experiment procedure of this study

In this study, the *n*‐back task was used to measure WM performance. Subjects were shown a continuous sequence of stimuli and were asked to indicate if the current image was the same or different from the previous stimulus (1‐back) or two stimuli prior (2‐back; Figure 1). The stimuli were 12 capital letters (A to L) that would appear at one of 12 positions, each of which was at the tip of one of six equidistant radii of an imaginary circular array, 2 or 6 cm from the screen center. The stimulus occupied between 3º and 4.5º of the visual angle on either side of the visual midline. The yellow stimuli were presented against a black background on a computer screen (AOC 17‐in. LCD monitor), with horizontal and vertical visual angles less than 5°. Participants were seated in an electrically isolated, sound‐ and light‐attenuated room, and viewed the computer monitor from a distance of 60 cm.

In this study, the verbal and spatial *n*‐back (1‐ and 2‐back) tasks involved identical stimuli and were distinguished only by the task demands, in that different instructions were provided to the participants before the beginning of each task. In the verbal task, participants were instructed to only remember the name of the letter, while in the spatial task, participants were required to remember its location only. All trials began with a “+” fixation, which remained on the screen for a random time interval ranging from 200 ms to 400 ms, then a capital letter serving as the target stimulus was presented for 300 ms. In the *n*‐back tasks, participants denoted match/non‐match by pressing a key. Half of the participants were instructed to press the “F” key with their left index finger to indicate same stimuli and the “J” key with their right index finger for different stimuli. For the remaining participants, the assignment of the response hand was reversed. In both the 1‐ and 2‐back tasks, match and nonmatch stimuli each occurred in 50% of the trials. If no response was provided, the next stimulus would appear after 1,500 ms.

## 4 ERP RECORDING AND DATA ANALYSIS

Electroencephalography (EEG) data were recorded from 64 scalp sites (10/20 system) using Ag/AgCl electrodes mounted in an elastic cap (Neuroscan Inc.; Sterling, VA; https://compumedicsneuroscan.com/quik-cap-electrode-system/, RRID: SCR_015817). The physical reference electrode was placed approximately 2 cm posterior to Cz. The electrode impedances were maintained below 5 kΩ. The vertical and horizontal electrooculography (EOG) data were recorded from above and below the left eye and from the outer canthi of both eyes, respectively. The EEG and EOG data were continuously recorded at a sampling rate of 500 Hz, applying a bandwidth filter of 0.05–100 Hz. The RT and the detailed responses of each trial were also recorded online.

The EEG data were referenced online to the left mastoids and offline re‐referenced to the average of the left and right mastoids (M1 and M2). Ocular artifacts were removed from the EEG signal using a regression procedure implemented in the CURRY software (Compumedics Neuroscan; Charlotte, NC; https://compumedicsneuroscan.com/products/by-name/curry/, RRID:SCR_009546). The ERP data were digitally low‐pass filtered at 30 Hz and epoched into periods of 1,200 ms, from 200 ms before to 1,000 ms after the onset of each target stimulus. Trials with various artifacts were rejected, with a threshold of ±75 μV. Waveform averages for each individual were calculated.

The following six sites were selected for the LPP and P2 statistical analyses at anterior electrodes: F3, FC3 (left); FZ, FCZ (midline); and F4, FC4 (right). The following seven sites were selected for the N1 statistical analysis at posterior electrodes: P3, PO3, (left); PZ, POZ, OZ (midline); and P4, PO4 (right). The electrode sites selected for analysis were based on the scalp distributions of the current data. Previous research has suggested that N1 is located over the posterior area, while P2 and the LPP are located over the frontal area (Luck & Kappenman, 2011; Zhang, Xie, He, Wei, & Gu, 2018). The time windows for the posterior N1, anterior P2, and anterior LPP were 140–200, 160–300, and 450–650 ms, respectively.

### 4.1 Statistical analysis

Data were analyzed using SPSS version 20 (IBM Corp.; Armonk, NY, https://www.ibm.com, RRID: SCR_002865) for Windows. SpO_2_ data of the two groups were compared using a two‐tailed two‐sample *t* test. To analyze the accuracy rate and RT of the two groups, a mixed‐model analysis of variance (ANOVA) with two factors was conducted, where the independent variables were “memory load” (1‐back/2‐back) as the within‐subjects factor and “altitude group” (HA/LA) as the between‐subjects factor. A repeated‐measures ANOVA with three factors (altitude: HA/LA; memory load: 1‐back/2‐back; electrode locations: left/midline/right) was performed for each ERP component under both the verbal and spatial tasks. Finally, the Greenhouse‐Geisser correction was used when the data violated the sphericity assumption.

## 5 RESULTS

### 5.1 Behavioral results

**Figure 2:**
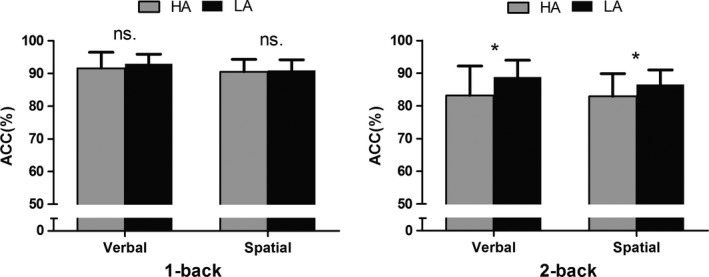
Accuracy rate of the verbal and spatial tasks. Note: *p < 0.05

Considering the accuracy rate in the verbal task, the ANOVA revealed significant main effects of group [*F* (1, 38) = 4.830, *p* < 0.05] and load [*F* (1, 38) = 37.718, *p* < 0.001]; specifically, that the accuracy rate of the HA group was significantly lower than that of the LA group and the accuracy rate in the 1‐back task was significantly higher than in the 2‐back task. The interaction between task load and group was significant [*F* (1, 38) = 4.570, *p* < 0.05], with a lower accuracy rate in the HA group than the LA group in the 2‐back task (*p* < 0.05) (Table 1, Figure 2).

**Table 1:** Mean and standard deviation of response time (RT) and accuracy rate (ACC) (*M* ± *SE*)

	Task type	Group	RT (ms)	ACC （%）
1‐back	Verbal working memory (WM)	HA	646 ± 88	91.6 ± 4.89
		LA	605 ± 62	92.9 ± 2.97
	Spatial WM	HA	585 ± 91	90.5 ± 3.79
		LA	555 ± 56	90.9 ± 3.24
2‐back	Verbal WM	HA	777 ± 145	83.2 ± 9.04
		LA	713 ± 148	88.8 ± 5.19
	Spatial WM	HA	719 ± 129	82.9 ± 6.95
		LA	700 ± 141	86.5 ± 4.52

Similarly, the spatial‐task ANOVA revealed a significant main effect of load [*F* (1, 38) = 48.054, *p* < 0.001], showing that the accuracy rate in the 1‐back task was significantly higher than that in the 2‐back task. The interaction between load and group was marginally significant [*F* (1, 38) = 3.514, *p* = 0.069], with a lower accuracy rate in the HA group than in the LA group on the 2‐back task (*p* = 0.055) (Table 1, Figure 2).

**Figure 3:**
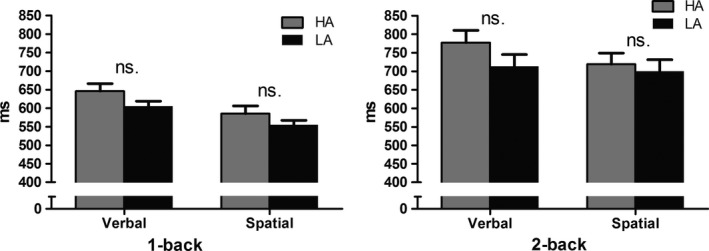
Response times of the verbal and spatial tasks

Considering RT, the main effect of load in the verbal task was significant [*F* (1, 38) = 38.411, *p* < 0.001], with a longer RT in the HA group than the LA group (Table 1, Figure 3). Moreover, the main effect of load in the spatial task was significant [*F* (1, 38) = 82.905, *p* < 0.001], with a longer RT in the HA group than in the LA group (Table 1, Figure 3). No other main or interaction effects were significant.

## 6 ERP RESULTS

### 6.1 ERP components in the verbal tasks

**Figure 4:**
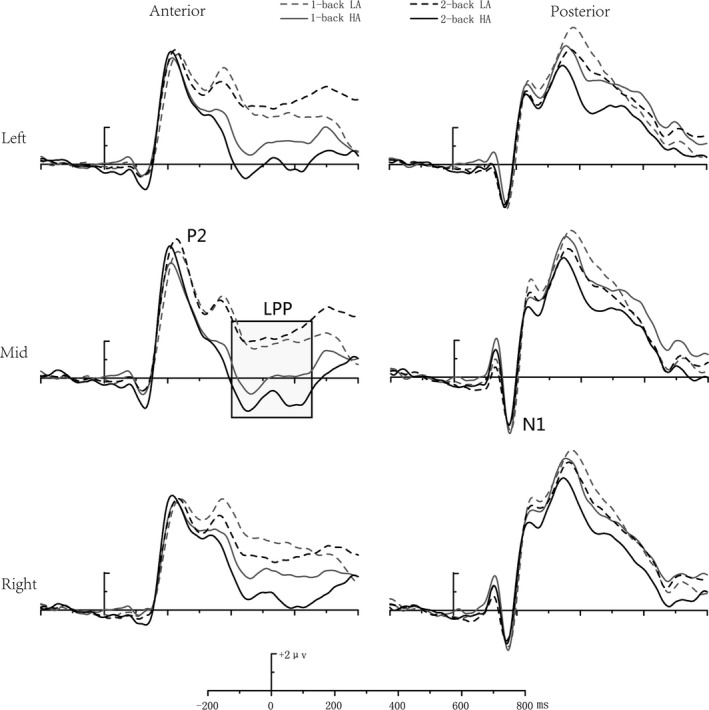
The event‐related potential component map of the verbal task condition

With respect to the mean amplitude of the anterior LPP component, the main effect of group was significant [*F *(1, 39) = 7.700, *p* < 0.01], with a smaller LPP amplitude in the HA group than the LA group. The main effect of task load [*F *(1, 39) = 3.407, *p = *.073] was marginally significant, with a larger LPP amplitude in the 1‐back than the 2‐back task. The main effect of site [*F *(2, 39) = 12.236, *p* < 0.001] was significant, with a larger LPP amplitude at the left and right prefrontal than midline sites (Figure 4).

The interaction effect between task load and group was significant [*F* (1, 39) = 5.275, *p* < 0.05], with a smaller LPP amplitude in the 2‐back task for the HA group than the LA group. No other significant main effects or interactions were found.

### 6.2 ERP components in the spatial tasks

By analyzing the amplitude of the posterior N1 component, the main effect of site was found to be significant [*F* (2, 39) = 14.776, *p* < 0.001], with a more negative N1 amplitude at right and left than at midline sites.

**Figure 5:**
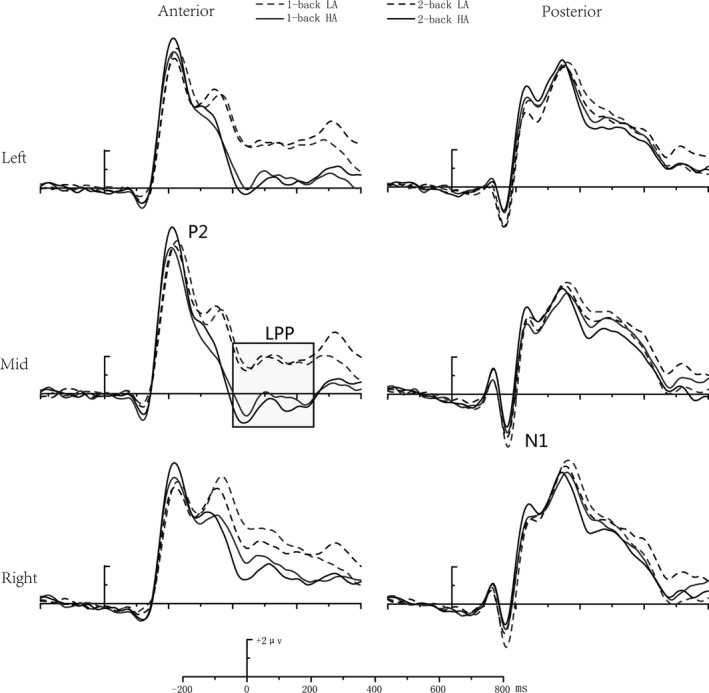
The event‐related potential component map of the spatial task condition

The amplitude of the anterior P2 component was found to have a significant main effect of site [*F* (2, 39) = 5.954, *p* < 0.01], exhibiting a larger P2 amplitude at left and midline than at right sites. The interaction between task load and group was also significant [*F* (1, 39) = 6.167, *p* < 0.05], with a larger P2 amplitude for the HA group than the LA group in the 2‐back task (*p* < 0.05) (Figure 5).

Regarding the mean amplitude of the anterior LPP component, the main effect of group was significant [*F* (1, 39) = 5.619, *p* < 0.05], with a smaller LPP amplitude in the HA group than in the LA group. The main effect of site [*F* (2, 39) = 14.343, *p* < 0.001] was also significant, showing a larger LPP amplitude at right prefrontal than left prefrontal and midline sites. The interaction effect between task load and group was significant [*F* (1, 39) = 5.135, *p* < 0.05], with a smaller LPP amplitude in the 2‐back task in the HA group than in the LA group (*p* < 0.05).

## 7 DISCUSSION

This study aimed to identify the impact of HA exposure on WM verbal and spatial components. Verbal and spatial WM performances were measured using the *n*‐back task, combined with ERP recording in an HA and an LA group. We found that (1) the accuracy rate in the HA group was significantly lower in both the verbal and spatial 2‐back tasks, but not in the 1‐back tasks; (2) the amplitude of the anterior P2 component was larger in the HA group in the spatial 2‐back task; (3) the amplitude of the frontal LPP component was smaller in the HA group in the verbal and spatial 2‐back tasks, but not in the 1‐back tasks.

The influence of HA exposure on matching procedures differed between verbal and spatial WM. In the spatial 2‐back task, a larger P2 amplitude was found in the HA group than in the LA group. According to previous studies, the frontal P2 component reflects a top‐down process, matching sensory inputs to memory representations (Federmeier & Benjamin, 2005; Freunberger, Klimesch, Doppelmayr, & Höller, 2007; Luck & Hillyard, 1994; Zhang et al., 2018); this is represented by a rise in P2 amplitude in the early selection process (Näätänen, 1990). In our study, the larger P2 amplitude in the HA group suggests that the early feature detection process for the spatial attributes of the stimuli were affected by the stress of HA, indicating the HA group may have incurred higher cognitive deficits to process the matching procedure in spatial WM updating. However, we did not find an effect of HA on P2 in the verbal WM tasks, suggesting a lack of influence of HA on the top‐down matching procedure for the physical attributes of the stimuli.

The maintenance processes of the HA group were affected in both the verbal and spatial WM tasks. In the behavioral results, the response accuracy in both the verbal and spatial WM tasks was significantly lower in the HA than the LA group. This finding shows that HA exposure might affect performance accuracy rather than processing efficiency. In previous studies, decreased accuracy rate was found in a verbal, but not spatial WM task (Yan et al., 2011a, 2011b). In modest concordance with these results, we found a decreased accuracy rate in both the verbal and spatial 2‐back tasks. The partial discrepancy might be attributable to several reasons. First, previous studies examined immigrants who were born and raised at HA, whereas the participants in our study were born and raised at LA and emigrated to HA in adulthood. Second, although the subjects recruited in this study had resided at HA for only 3 years, the impact of HA on verbal capabilities might be reflected in their verbal WM. Considering that the two groups did not have significant differences in RT, but demonstrated a significant difference in performance accuracy rate, it is possible that some subjects in the HA group might have made a trade‐off between accuracy rate and RT.

Regarding the ERP results, the frontal LPP component showed lower amplitude in the HA group than the LA group in both the spatial and verbal 2‐back tasks. According to Ruchkin et al. (1990), the LPP may reflect information maintenance in a WM task. The reduced LPP amplitude in the HA group may indicate an impaired effort to maintain representation. Reportedly, the LPP is sensitive to the attentional demands of WM tasks (Corbetta et al., 1993; Owen et al., 2010; Yantis et al., 2002) and the amplitude of the LPP is smaller when attention is focused on an additional mental activity (Gevins et al., 1996; Mcevoy, Smith, & Gevins, 1998). Therefore, the significant attenuation of the frontal LPP amplitudes of both the spatial and verbal 2‐back tasks in the HA group may suggest that attentional resources might have been consumed by previous cognitive processing and that the remaining available resources for the maintenance process were reduced.

The influence of HA on WM was affected by perceptual load. In this study, we found increased P2 and attenuated LPP amplitudes in the HA group in the 2‐back task, but not the 1‐back task. According to the cognitive load theory, limited WM capability leads to difficulty in processing information from multiple sources, and when there is simultaneous influx of a variety of information, the cognitive load will increase (Sweller, 2010). According to our previous study (Wang et al., 2014), the HA group required more cognitive resources to complete the higher perceptual load task. The increased P2 component of the HA group in the 2‐back task may indicate that more cognitive resources were used in the matching process, and that insufficient resources remained for the maintenance process, explaining the smaller LPP found in the HA group. Moreover, the difference between the 1‐back and 2‐back tasks be a result of task difficulty and strategies (Sandrini, Fertonani, Cohen, & Miniussi, 2012; Sprenger et al., 2013). WM strategies may depend on cognitive load, in that 1‐back tasks may rely on the familiarity of the stimuli, but 2‐ or 3‐back tasks might be primarily based on refresh strategies (Sprenger et al., 2013). Notably, refresh strategies require more WM resources to maintain the successive order of the stimuli and refresh this order after the arrival of new stimuli (Lovett, Daily, & Reder, 2001).

In this study, we did not find an influence of HA on N1 in verbal or spatial WM, suggesting that HA does not impact early attention to the physical and spatial attributes of the stimuli. As N1 is typically considered to reflect automatic processing (Näätänen, 1990) and is associated with the early monitoring and feature analysis of the stimuli, the stimuli that require more attentional resources will induce greater N1 amplitudes (Näätänen & Picton, 2010).

A limitation of our study is that although the participants physically adapted HA, physical data such as ventilation rate and heart rate were not recorded. These data should be considered in future studies to ensure they are compatible with the experimental condition. Moreover, while hypoxia is the most important impact factor in HA areas, we did not consider other possible relevant factors such as climate or culture. For these reasons, the results must be cautiously interpreted.

## 8 CONCLUSION

In this study, the ERP method was used to explore the spatial and verbal WM performance of individuals who migrated from LA to HA. We found that the matching process was influenced by HA exposure only in the spatial 2‐back WM task, while maintenance processes were influenced in both the spatial and verbal 2‐back tasks. Our results suggest that HA influences spatial and verbal WM via different mechanisms, while the impairment of spatial WM may involve both matching and maintenance processes, the deficit in verbal WM may only be due to an impaired maintenance process.
